# Mental healthcare for asylum-seekers and refugees residing in the United Kingdom: a scoping review of policies, barriers, and enablers

**DOI:** 10.1186/s13033-021-00473-z

**Published:** 2021-06-14

**Authors:** Teresa Pollard, Natasha Howard

**Affiliations:** 1 London School of Hygiene and Tropical Medicine, Department of Global Health and Development, 15-17 Tavistock Place, London, WC1H 9SH UK; 2 Saw Swee Hock School of Public Health, National University of Singapore, 12 Science Drive 2, Singapore, 117549 Singapore

**Keywords:** Mental health, Health policy, Asylum-seekers, Refugees, UK

## Abstract

**Background:**

Since 2011, a large influx of asylum-seekers and refugees has put pressure on the UK’s under-resourced national health services and mental health services. Asylum-seekers and refugees (ASR) may experience traumatic events pre-departure, life-threating circumstances on their journeys, and difficulties integrating into host countries related to immigration policies, social isolation, poor living conditions, and unemployment, all of which can significantly affect their mental health. This topic is increasingly important due to the numbers of people seeking asylum and growing concern for their mental health on resettlement. This study examined UK-wide policies and guidance, healthcare practices, barriers, and enablers of mental healthcare for ASR residing in the UK.

**Methods:**

We conducted a scoping review using Arksey and O’Malley’s 2005 framework, which included semi-structured interviews with stakeholders from non-governmental organisations, academia, UK National Health Service, and community groups. We synthesised and analysed literature and interview data thematically to examine current barriers and potential enablers of ASR mental health support in the UK.

**Results:**

We included 39 literature sources, of 1,638 identified, and 10 stakeholder interviews**.** Sources, most published in 2019 (n = 13), included data from England (n = 13), Scotland (n = 3), Wales (n = 3), and Northern Ireland (n = 2) and covered access to care (n = 16), mental health disorders (n = 7), impacts on health (n = 7), barriers to care (n = 13), policies and plans (n = 4), and clinical recommendations (n = 3). Synthesised themes from literature and interviews included existing barriers (i.e. communication difficulties and lack of funding, resources, and political will) and potential enablers (i.e. proposed provision practices, social needs of ASR, and policy changes).

**Conclusions:**

There is a gap in the literature regarding UK-wide assessment of access and delivery of mental healthcare for ASR in the UK. Time sensitive and culturally appropriate approaches are needed, with greater funding and resource support from the UK Government. This study provides justification for a call to relax hostile environment policies, and for ASR-specific mental health services and support to be considered within the UK. Further research is needed to assess implementation of guidelines across the UK.

**Supplementary Information:**

The online version contains supplementary material available at 10.1186/s13033-021-00473-z.

## Background

Since the Syrian conflict began in 2011 there has been an increase of migrants into Europe and the United Kingdom (UK) [1]. People have always fled conflict in hope of finding safety for themselves and their families [2]. The number of asylum-seekers and refugees (ASR) has continued to rise. From about 2015, a large influx of people fleeing conflicts in Syria, Afghanistan and Iraq became known as “Europe’s refugee crisis” [3]. The 1951 Refugee Convention defines a refugee as someone “unable or unwilling to return to their country of origin owing to a well-founded fear of being persecuted for reasons of race, religion, nationality, membership of particular social group, or political opinion”[4]. In reality, to be formally recognised as a refugee, a migrant must first be given asylum. Asylum- seekers, defined as “someone who has arrived in a country and asked for asylum” do not yet hold refugee status or documentation [5]; and in many countries, such as the UK, they have fewer rights than citizens or refugees [6]. European countries have faced considerable pressures to accept large numbers of asylum-seekers and not all have complied [7]. According to UK Home Office statistics, of 34,354 asylum applications in 2019, 11,596 were granted, and 5,606 were granted protection through resettlement schemes [8]. Such figures highlight the significant numbers of people in the UK with accepted or failed asylum applications who may need additional support.

World Health Organisation (WHO) technical guidelines describe potential stressors and mental disorder risks for ASR, including distressing events prior to departure (e.g. conflict and violence), distressing incidents during travel, environmental factors in host countries (e.g. poor living conditions, integration difficulties, unemployment) [9]. Complex legal procedures required to claim asylum, obtain permission to reside within the country, and enduring the claim consideration process can cause significant additional stress [10, 11]. Post-traumatic stress disorder (PTSD) is common among ASR [12], with up to 31% prevalence for many years after immigrating [13]. Research found prevalence of depression up to 31% and anxiety up to 11% amongst ASR, with anxiety particularly increased soon after migration [13]. The UK government recognises increased risk of mental disorders among ASR in its ‘Mental health: migrant health guide’, which estimated an increased 5–10% risk of mild or moderate mental disorders, including depression, anxiety, and PTSD and suggested a large proportion of migrants entering the UK are at risk [14]. The WHO Health Evidence Network synthesis report [15] outlines barriers for ASR accessing mental healthcare including: difficulty understanding or navigating the healthcare system and services, little knowledge regarding entitlements to healthcare in the host country, difficulty communicating due to language barriers, and decreased trust of foreign healthcare providers and organisations. The complexity of these barriers requires guidelines to support ASR with effective and consistent care [15]. The European commission-funded WHO guidance for mental health promotion and care for refugees advocates eight priority action areas; including providing interpreters within services, clearer guidelines on ASR healthcare entitlements, adequate training of mental health staff to work with vulnerable groups such as ASR, and further long-term evidence- based research and evaluation of services [10]. These areas are vital, considering the prevalence of mental disorders and complexity of barriers to care, but not all countries actively implement them.

### UK policies and legislation

The UK Home Office oversees immigration laws and policies for England, Wales, Scotland, and Northern Ireland, but health policies are devolved so legal frameworks for immigration and service accessibility may differ [16]. The UK Home Office stipulates that migrants who flee their country of origin and travel to the UK in hope of resettling may seek asylum, which allows them “indefinite leave to remain” [17]. Migrants should apply as soon as they reach the UK border. The process is complex and slow, requiring an average of 6-months investigation and interviews from immigration officers [18]. Those approved for refugee status are eligible for UK refugee protection rights (1967), based on Article 14 of the Universal Declaration of Human Rights [4]. Refugees can enter into The Gateway Protection Programme managed by the Home Office, but it only accepts 750 refugees per year [19]. The Mandate Refugee Programme accepts those who qualify for protection under UNHCR criteria, but do not seek asylum within the UK. The Syrian Vulnerable Person Resettlement programme [20], supported by the Refugee Council, aimed to allow 20,000 Syrians into the UK by 2020, prioritising those at risk or in need of medical care [19].

Refugees resettled by the Home Office have reception support and arrangements organised by local authorities, which may be coordinated by regional migration partnerships [16]. Therefore, information about healthcare and other service providers is provided by workers assigned to resettled families [16]. ASR rely on charities and community groups, highlighted by their assigned caseworkers, for help completing registration forms and accessing healthcare. Asylum-seekers rely on government support for living costs, currently cash support of £35.39 per person per week for food, sanitation and clothing [21]. By contrast, unemployed British citizens receive job- seekers allowance of up to £73.10 per week [22]. However, undocumented migrants or refused asylum- seekers have no access to public funds and cannot open bank accounts or find employment [23]. Financial assistance gaps are left to charities such as Freedom From Torture, the British Red Cross, and the Refugee Council to fill [24].

### Healthcare access for overseas visitors

*In England*, primary care, including GP consultations and treatment, is free of charge. However, refused asylum-seekers or undocumented migrants are charged for secondary care [25]. In 2017, a new government regulation was introduced, whereby all hospitals were legally required to check patient eligibility for free national health system (NHS) healthcare. Patients must now pay upfront before receiving treatment if they cannot prove their eligibility, unless it is ‘urgent’ or ‘immediately necessary’ [26]. This is also a requirement for NHS community health services, including mental health services, with those detained under the Mental Health Act 1983 [27] theoretically exempt from treatment charges [28].

*In Wales,* both primary and secondary healthcare are free-of-charge for overseas visitors, including undocumented migrants and asylum-seekers. The NHS Amendment Wales Regulations [29] indicates that refused asylum-seekers can access free healthcare in Wales. However, primary care providers decide whether overseas visitors may be accepted as an NHS patient or privately for non-emergency treatment. For secondary care, charges ‘may occur’ [29].

*In Scotland,* primary and secondary healthcare is free-of-charge for ASR as for any resident, and undocumented migrants or refused asylum-seekers are not to be charged [30]. The Scottish refugee policy entitles anyone who has made a formal application for asylum, whether pending or unsuccessful, to treatment on the same basis as a UK national ordinarily resident in Scotland [31].

*In Northern Ireland,* healthcare eligibility guidelines are similar to other regions, but not all migrants are eligible for free GP primary care. For example, undocumented migrants are liable to charging for primary and secondary care, with the exception of Accident & Emergency (A&E) treatment or compulsory detention. From 2015, refused asylum-seekers have the same entitlements as other residents [32].

### Mental health policies and plans generally lack explicit guidance on ASR

*England*’s 2011 “No Health Without Mental Health: A cross-government mental health outcomes strategy for people of all ages” [33], targeted improved outcomes for people with mental health problems through high-quality services equally accessible to all [31]. Further mandates, including in 2015, have recognised mental health as ‘on par’ with physical health within NHS England. Mental health policy includes the Five-Year Forward View for Mental Health [34], which commits to working towards a more equal response across mental and physical health and expanding access and waiting time standards by 2020–2021. In 2017, the Mental Health Act 1983 was revised to address disproportionate numbers of people form black and minority ethnic groups detained [31], but does not highlight ASR.

*Wales* has “Together for Mental Health: a strategy for mental health and wellbeing in Wales 2012” [35], and the 2019–2022 delivery plan, outlining the needs of vulnerable groups including ASR [36].

*Scotland’s* Mental Health Strategy 2012–2015 [37] sets out government priorities and commitments to improve mental health services and prevent mental illness. In 2014, a Scottish bill was launched to help people with mental disorders access effective treatment quickly and easily. In 2018, the Scottish government published “Every life matters”, a suicide prevention action plan, but does not specifically mention ASR mental health.

*Northern Ireland’s* regional mental healthcare pathway, “You in mind” 2014 [38], commits health and social care services to deliver care that is more personalised and improves experiences of people with mental health problems [31]. However, guidelines do not specifically refer to care for ASR.

### Aim

This scoping review aimed to examine existing UK-wide health policies, practices, barriers, and enablers for mental healthcare for ASR residing in the UK. The importance of this study is in describing the current policy environment for ASR access to mental healthcare in the UK and examining mental health services provision for this vulnerable group, which can inform mental healthcare policy and practice for ASR in the UK.

## Methods

### Study design

We conducted a scoping review, using Arksey and O’Malley’s six-stage framework: (i) identifying the research question, (ii) identifying relevant sources, (iii) selecting sources, (iv) charting data, (v) collating, summarising, and reporting results, and (vi) stakeholder consultation [39]. Scoping reviews provide a broad overview, which is particularly useful for new or under-researched topics, while stakeholder consultation can be used to add rich experiential data from those actively engaged in the field.

### Stage 1: Identifying research question

Our research question was: ‘*What is the scope of existing literature on mental healthcare for ASR residing in the UK and what are key barriers and enablers in UK-wide health policies, guidance, and practice*?’.

### Stage 2: Identifying sources

TP searched seven electronic databases systematically (i.e. OVID Medline and a complimentary search of PubMed; OVID PsychINFO; EThOS; Ovid Global Health; Ovid EMBASE; BASE) plus, Google Scholar (first 10 pages) and APA PsychEXTRA for grey literature. Eligible sources were English language original research published between 2011 and 2020. Subject headings were used in conjunction with key words, with search terms used across databases checked against MeSH terms to ensure related terms were included. The following terms were adapted depending on the database: Mental Health OR (mental health or wellbeing or psychological* state or psychological condition or mental state), AND "migrants and immigrants"/ or undocumented immigrants/ or refugees/ or "transients and migrants"/ OR (Refugee* or migrant* or asylum seeker* or immigrant*), AND united kingdom/ or England/ or northern Ireland/ or Scotland/ or Wales/ OR (United Kingdom* or UK or Wales or Scotland or Ireland or England). We used forward and backward citation searching to identify further relevant sources not captured in the original search.

### Stage 3: Selecting sources

TP screened sources against eligibility criteria (Table 1), first by title and abstract then full text review, with screened sources reviewed by NH for inter-rater reliability. All sources reporting primary or secondary research data (i.e. quantitative, qualitative, systematic review) were eligible for inclusion if data were collected prior to 2011, based on UK evidence, and included relevant topics. 2011 was used as a cut-off to enable focus on policies relevant to the European ‘refugee crisis’ and Syrian conflict. To capture additional relevant sources, the term ‘refugee’ was expanded to include asylum-seekers, and migrants. Only England, Scotland, Wales, and Northern Ireland were included. Sources not relating to national policies, mental healthcare services, and resources, and ASR were excluded.

**Table 1:** Eligibility criteria

Criteria	Included	Excluded
Publication year	2011–2020	Before 2011
Language	English	All other languages
Countries	England, Wales, Northern Ireland, Scotland	All other countries
Theme	Mental health services for ASR in the UK Mental health risk factors, needs, and outcomes from residing in the UK Delivery of mental healthcare Measures used to assess mental healthcare delivery for ASR Policies referring to access to and provision of mental healthcare	All other themes Not about migrants residing in the UK
Publication type	Thesis Agency reports Journal articles Book chapters Conference abstracts	Media articles Conference abstracts for which an article existed

### Stage 4: Charting data

TP charted data using the following fields: lead author, publication year, country, aims, methods, population, and key findings (Additional file 1). NH reviewed the process.

### Stage 5: Collating, summarising and reporting results

We synthesised literature data thematically, using Braun & Clarke’s six-stage method[40]. First, TP read and became familiarised with the data. Second, TP generated initial codes manually. Third, TP and NH developed a coding structure iteratively, and TP collated codes related to barriers and enablers into preliminary themes. We examined relationships between codes, compiled them, and summarised contents of each theme, comparing codes and initial themes for both literature and interview data. Fourth, we reviewed initial themes across literature and interview data, splitting, combining, or discarding less meaningful ones as appropriate. Fifth, we defined and named final themes through discussion and further integration. Finally, we refined and contextualised themes during the reporting process (Additional file 2).

### Stage 6: Consulting stakeholders

TP conducted semi-structured interviews in 2019 with ten stakeholders from NGOs, academia, UK National Health Service, and local community groups. Recruitment was initially purposive followed by snowballing. We circulated recruitment emails through universities and organisation networks, which yielded 10 participants. The topic guide, developed from the literature, included their work with ASR, opinions on available services for mental health and migrants, any new approaches needed, barriers and challenges, and relevant policies and laws. TP obtained written informed consent prior to interview, which were conducted by phone or Skype and recorded using Microsoft Windows audio-recorder app, and transcribed audio files.

We conducted thematic analysis of interview transcripts, using Braun & Clarke’s six-stage method as described in Stage 5[40]. In summary, TP conducted phases 1–2 separately on the two datasets. In phase 3, we compared codes and themes for both datasets, which enabled us to synthesise themes through inductive consolidation and discussion during phases 4–6.

### Ethics

Ethics approval for interviews was provided by the LSHTM MSc Research Ethics Committee (reference 16,930).

## Results

### Scope, nature, and distribution of literature

**Fig. 1:**
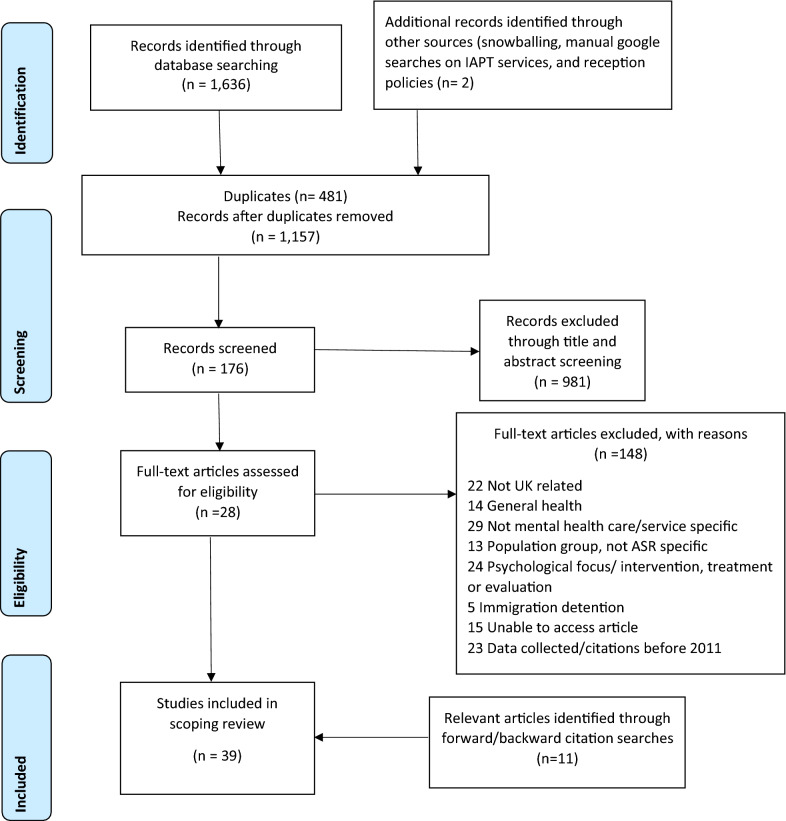
PRISMA flowchart. Preferred reporting items for Systematic reviews and Meta-Analyses (PRISMA) diagram

We included 39 literature sources of 1,638 identified (Fig. 1). Table 2 shows initial themes, categorised by source type and lead author. Source types included 24 journal articles (62%), 7 technical reports (18%), 2 theses (5%), and 6 commentaries and editorials (15%), most published in 2019 (n = 13). Sources included general UK data (n = 17) or data specific to England (n = 14), Scotland (n = 3), Wales (n = 3), or Northern Ireland (n = 2). Main topics covered were access to care (n = 23), barriers to care (n = 15), policies and plans (n = 10), mental health disorders (n = 10), impacts on health (n = 7), and clinical recommendations (n = 4).

**Table 2:** Themes by source type, alphabetised by lead author

Lead author (year)	Theme					
	Access to care	Psychological disorders and mental health	Policy and plans	Clinical recommendations	Health impacts	Barriers to care
Journal article (N = 24)	N = 15	N = 6	N = 2	N = 3	N = 7	N = 12
Brandenberger (2019) [48]	✓				✓	✓
Brenman (2020) [84]	✓					
Chiarenza et al*.* (2019) [69]	✓					✓
Cowles (2019) [68]				✓		
Fang et al*.* (2015) [76]		✓				
Hiam et al*.* (2019) [67]	✓					✓
Jannesari (2019) [43]	✓	✓			✓	
Juárez (2019) [73]			✓		✓	
Kang (2019) [46]	✓					
Majumder (2019) [53]	✓					✓
Majumder et al*.* (2019) [85]	✓					✓
Majumder et al*.* (2015) [64]]					✓	✓
Murphy et al*.* (2020) [66]	✓					✓
Murphy et al*.* (2017) [75]	✓					✓
Piacentini (2019) [49]	✓				✓	
Poduval et al*.* (2015) [54]	✓				✓	✓
Priebe et al*.* (2016) [57]		✓	✓			
Quinn (2014) [60]		✓			✓	✓
Riza et al*.* (2020) [79]				✓		
Robertshaw (2017) [61]	✓			✓		✓
Sanchez-Cao et al*.* (2013) [78]	✓	✓				
Satinsky et al. (2019) [62]	✓					
Strang (2019) [63]		✓				
Tesfaye et al*.* (2015) [83]						✓

Thesis (N = 2)	N = 1	N = 2	N = 0	N = 0	N = 0	N = 0
Mohamed (2012) [45]		✓				
Rae (2014) [59]	✓	✓				

Report (N = 7)	N = 6	N = 1	N = 5	N = 0	N = 0	N = 1
Doctors of the world (2015) [55]	✓		✓			
Karamanidou et al*.* (2020) [16]			✓			
Nellums et al*.* (2018) [24]	✓					
Nellums et al*.* (2018) [50]	✓					
Patel (2017) [52]	✓		✓			
Taylor (2018) [41]	✓		✓			
Viner (2018) [71]	✓	✓	✓			✓

Other (N = 6)	N = 1	N = 1	N = 3	N = 1	N = 0	N = 2
Brooks (2019) [72]		✓				
Cox (2020) [77]			✓			
Crawshaw et al. (2018) [82]				✓		
Hiam et al*.* (2018) [42]			✓			
McKeown et al*.* (2020) [44]			✓			✓
Waterman et al*.* (2020) [28]	✓					✓
Total: N = 39 (%)	23 (60)	10 (26)	10 (26)	4 (10)	7 (18)	15 (38)

Additional file 1 shows research methods included qualitative interviews and focus groups (n = 15), undescribed (n = 7), mixed-methods (n = 7), reviews (n = 6), or case studies (n = 4). Study population sizes ranged from a case study of 1 refugee up to 849 ASR, with most (n = 16) including study populations of under 20 people. Twelve sources focused on particular ASR populations (i.e. from Sri Lanka, Afghanistan, Somalia, Iran, Iraq, Eritrea). Twelve sources focused on the mental health of particular groups, i.e. child and adolescent refugees or unaccompanied minors (n = 8), refugee men (n = 3), and refugee women (n = 1).

### Interviewee characteristics

Table 3 shows the characteristics of 10 interviewees based across England, including psychotherapists, psychiatrists, link workers, and academics. Most interviewees worked for the NHS (n = 5) and community charities (n = 4).

**Table 3:** Interviewee characteristics

ID	Sector	Job role
i1	National Health service (NHS), Academic/research (AR)	Consultant psychotherapist
i2	Local community group/Charity (LCG)	Counselling service manager
i3	GP practice, NHS	Link worker
i4	NHS	Retired consultant psychiatrist
i5	Local community group/Charity (LCG)	CEO and clinical director of counselling service
i6	Local community group/Charity (LCG), Non-governmental organisation (NGO)	Support coordinator and advisor
i7	NHS, Non-governmental organisation (NGO)	Consultant psychiatrist and honorary lecturer
i8	Non-governmental organisation (NGO)	Psychotherapist and clinical service manager
i9	Academic/research (AR), NHS	Honorary lecturer and consultant psychiatrist
i10	Local community group/Charity (LCG)	Project manager

### *Thematic findings*

We grouped inductive themes from literature and interview data as either barriers or enablers. Barriers were: (i) hostile environment, (ii) lack of political will and resources, (iii) communication difficulties, (iv) service delivery inconsistencies, and (v) fear, trust, and uncertainty. Enablers were: (i) proposed provision approaches, (ii) social support for mental health, and (iii) supportive policies.

### Hostile environment barriers

Although we identified relatively little research in the literature, the UK Government has been criticised for creating a hostile environment for migrants through restrictions on immigration, reducing trust, and people becoming fearful of data sharing[41, 42]. Jannesari found ASR expressed feelings of “bureaucratic torture” about the asylum process [43]. This process, combined with ASRs’ often limited trust of ‘foreign’ (i.e. British) providers and absence of safe spaces, made recovery from migration stresses and trauma particularly difficult [44].

Interviewees were positive about their work with ASR but felt discriminatory and negative views about migrants were sometimes directed at them, and also led to deprioritisation of funding for migrant services. The narrative in the press from far-right groups was seen as stigmatising their work [42]:

“The far-right agenda which is gaining a consensus across the globe, which means migration in general is seen as a negative thing… I think the Brexit atmosphere is a negative thing and the hostile environment […]. Mental health anyway is a nightmare in this country in terms of provision for the general public…” (i8)

The hostile political environment reportedly filtered down to community levels [45].

“The hostile environment was prompted by Theresa May, as when she was home secretary, and I think if you look at the papers, they overwhelmingly feature the discourse that seems to be negative rather than positive. I think there's a sort of fight between people who take a more liberal humane view and those who will see refugees as chancers or scroungers…so I think that is a big struggle.” (i1)

Improvements rely on improved awareness and compassion, from people who can make decisions to improve services.

“The mental ill health of refugees isn’t necessarily having an impact on wider society. It’s a bit of a hidden problem, and I think unfortunately the needs of refugees are not political or voting priorities. Refugees are not a demographic with much influence in that regard.” (i10)

Services may be influenced by the hostile environment, and even within the NHS migrants have experienced discrimination from practitioners [46]:

“In that survey we did last year, there were quite a few signs of definite racist attitudes to people, discriminatory attitudes. I don’t think that’s the majority at all. I think the majority would definitely like to help but there's some really awful things happen to people saying refugees should go back to where they came from.” (i4)

### Lack of political will and resources as barriers

Literature and interviewees indicated lack of political will and resources were major challenges. Public opinion and political will were generally considered as negative towards ASR [41]. Interviewees outlined how mental health services were not a government priority and no mainstream services were available.

“…the reality is that people are feeling stretched, many people have been living with austerity for a very long time and when you're living with austerity and you are poor yourself it is very difficult to say that's fine take some of what I’ve got and give it to somebody else […] I’m not aware of the government doing anything about it because I don’t know it's a priority, in fact it’s probably the opposite…” (i5)

Allocation of resources to ASR was difficult as general mental healthcare remained insufficient for British citizens [16]. Equally, specific areas with larger refugee resettlements had more need of mental health provision than other areas of the UK [16].

“In the areas like central London where we see refugees all the time, attitudes are generally positive, but other areas of the UK maybe it’s not like that. ​They don’t have the long history of receiving migrants and refugees, with services experienced in delivery to this population...” (i9)

Currently NHS England invests 1.4 billion British pounds in mental health services, but no specific funding is allocated for ASR, nor are there specific guidelines for ASR mental health[47]. If a project needed extra funding, services must provide evidence to show patient improvements in their services. NGO and community interviewees reported working very effectively together despite lacking funding. Finance and money were identified as the main barrier to supporting ASR, including difficulties in feeling stretched with resources, time, and appointments.

“Public health is more concerned about infectious diseases with refugees, like TB, rather than mental health issues…” (i3)

### Communication difficulties as barriers

Literature showed communication to be an important healthcare need for ASR, as successful resettlement and psychological wellbeing depend upon language proficiency [48]. The literature indicated need for more effective dissemination and communication of information by the Home Office and healthcare providers [24]. Migrants described confusion over NHS structures and how to access healthcare or arrange appointments [46]. Asylum-seekers often have less knowledge about the health system as they may have less opportunity to access information beforehand than those migrating for employment [49].

Those with low literacy may depend on someone else, potentially reducing disclosure of sensitive but important information [24, 50]. Availability and quality of interpretation services is inconsistent across the UK and health-worker training in working with interpreters is necessary [51]. Importantly, a relationship of trust and mutual respect is needed between healthcare professionals, interpreters, and patients [48, 51].

All interviewees described language barriers, with variations in availability and quality of interpreters due to lack of funding and low professional standards. Languages provided by interpreter services were usually mainstream and lacked dialects. GPs had no training to work with interpreters [52].

“NHS [secondary care therapy] services don’t have any training for working with interpreters so quite bad practice can happen unintentionally.” (i4)

Some providers reportedly treated the need for interpretation services as a nuisance.

“The professional standards for contracting interpreters are hardly respected really because there is very little money allocated to the need for interpreting and is seen as a nuisance in the ‘main’” (i5)

Although many ASR speak English, reading and writing for some people may be weaker[24]. Many ASRs came from countries with low literacy rates, particularly among women [24, 50], and sometimes trauma can affect learning abilities.

“Cognitive capacities are quite often affected especially if you are on medication as well, plus a lot of the refugee and asylum-seekers we see come from poor countries and they may have only received education to what we would regard in this country as ‘primary school level’. So then being expected to learn a new language is a big ask when literacy levels aren’t great.” (i2)

Expressing complex emotions in an unfamiliar language is difficult, as is talking about trauma and psychological illness [53], particularly given the stigma surrounding mental health.

“It’s quite a big challenge to be able to express complex emotional feelings in another language, I think that requires some fluency and, in our experience, even when people have got a proficiency in English, they prefer to speak in their mother tongue.” (i2)

ASR who do access mental health services may not be aware they can request an interpreter [54].

“The clients we’re working with, refugees, they’re not always aware they have a right to an interpreter when they go for an appointment.” (i6)

### Service delivery inconsistencies as barriers

The literature acknowledged variation in service delivery. Despite government regulations and policy, each general practice or NGO decided the care they could provide. Doctors of the World (DOTW) researched access to GP registration in England in 2015 [55] and 2017 [52] and found the main barrier was providing paperwork for registration with a GP. Of the 849 GP registrations made by DOTW in 2015 [55], responses were inconsistent and 39% of cases were refused, compared with 20% in 2017 [52]. GPs are often unaware of current policies and entitlements surrounding healthcare for ASR, as asylum policies can be complex [24, 50]. Proof of identity or address are often wrongly demanded [23, 28], and rejection creates fears that data would be shared with the Home Office, although data sharing between Home Office and NHS has been abolished [41]. Healthcare staff rarely have the knowledge of immigration law required to review complex issues of patient eligibility [28].

Interviewees from different areas highlighted inconsistencies of care across the UK, particularly outside London and in non-urban areas of the UK [16]. Clinical commissioning groups (CCGs) are decision-makers for health services provision and resourcing across England [56]. CCGs commission healthcare services for their area through assessment of local needs (nhscc.org), and if they do not recognise the need for more support to ASR, they will not provide additional funding.

“It is often said that their mental health issues are circumstantial because they are going through the asylum process and that it's because they haven't got a visa… and if they’ve got specific issues say PTSD, caused by torture or something like that, then there's really no support…There's a real lack of support in our region.” (i6)

An interviewee discussed funding limitations in relation to the Syrian resettlement programme in one area of the UK:

“…for those on the Syrian refugee resettlement programme, I was told that their NHS or the CCG gets a fee of 2600 pounds for this person regardless of their age or their sex. The CCGs will choose 600 pounds of that to register the person with a GP and then maybe 2 basic blood test but the 2000 pounds disappear. So one of the things we’ve been trying to do with the CCG is ask about that and ask if we can use part of this 2000 to support their work in the clinic.” (i7)

Treatments offered by these services are usually short-term for people “who can be quickly recovered”[46], although evidence suggests migrants’ health may worsen over time meaning they cannot recover quickly and may need long-term support[57, 58]. However, one interviewee explained how there are a large number of professionals working in the area who really strive to help and provide the care ASR need:

“We’re very lucky to have a lot of surgeries signing up towards safe surgery initiative, which is basically not asking the person about their eligibility for treatment. They do ask about the refugee status whether they are asylum-seekers or refugees, but they wouldn't stop treatment if they are undocumented.” (i7)

### Fear, trust, and uncertainty as barriers

Literature and interviewees described ASR fears, particularly of stigma, and lack of trust in mental health providers [59, 60] as reducing mental health care-seeking [59, 60]. Interviewees described access as associated with ASR fears. Notions of trust and safety were highlighted in the literature and interviews [61–64]

“Clients will drop-out or not connect, because there needs to be a lot of work to build trust, and it should be sensitive in approach…” (i8)

Trust and confidence was vital to mental health service access and use [48]. Many ASR felt more trust in providers who “understood” their situation or were of a similar background, wanting more providers from ethnic minorities in psychiatric services [62], to build trust and help them integrate into a new environment [63]. Migrants come from culturally diverse backgrounds, and seeking help from strangers rather than family or friends may seem unfamiliar [65]. However, many recognised that stigma around mental health within communities of origin and diaspora could also increase ASR fears of accessing. [60, 65] and in some circumstances it might be preferable to speak with impartial strangers.

“Even in their own language it does not mean that things will be easy during the consultation, because there is a huge kind of degree of stigma regarding psychiatrist, mental illness, and the element in taking a package with medication.” (i7)

Literature and interviewees described ASR uncertainties in navigating the health system [24, 46]. Even ASR who do access mental health services may not be aware of their rights in the UK [54]. Many ASR may be deterred from accessing mental health services due to concerns over charging regulations and costs [66]. Uncertainties were worsened by providers who did not understand the immigration process and healthcare eligibility [24, 54]. Research confirms routine and primary healthcare use is lower among migrants than non-migrants and a high rate of A&E use among migrants, potentially due to confusion about the health system [46]. ASR are also relatively likely to be charged for seeking A&E care due to secondary care costs [50, 67].

“Navigating systems is a challenge for a lot of people that we work with. People don’t know what services are available. Even some of my colleagues who work in other teams that don't work specifically with refugees won’t be necessarily aware…” (i10)

Interviewees, however, reported that the numbers of ASR accessing their services largely related to the resources available to treat patients and the ability to reach clients in need of services. One interviewee noted that confident ASR would access services, but many were unaware of them or too unwell to seek help.

“There’s a lot of people who just stay at home. They’re too unwell to access us…” (i6)

### Proposed provision approaches as enablers

Several approaches noted in literature and interviews could enable improved mental health services provision for ASR, as evidence suggests some access barriers may be reduced by positive experiences of services [50]. For example, if ASR have a good experience accessing mental health services, this may increase help-seeking and trust in the health system [50]. Potential approaches included cultural competence [61], explicit consideration of therapeutic boundaries [68], and psychotherapeutic support for trauma [69].

Competence, particularly cultural competence, is of growing interest in ASR healthcare provision [61]. ASR experience many hardships adjusting to a new culture and the pressures of a new environment [9]. Such training could help mental healthcare providers, who may not have been trained to support ASR needs [61]. It might also increase consistency across providers.

“I think there's a really big issue about intercultural working. NHS staff are not generally adequately trained in racism, and the general needs of any cultural language group, people within the asylum system. Staff often have really limited knowledge about all the implications of that and all the implications of the stresses on people, the instability, the effects of what they might write on peoples’ notes.” (i4)

Culturally competent providers could potentially offer more nuanced support or work to develop culturally-relevant approaches. Mental health and counselling support are Western concepts and current UK practices are not generally aligned to non-Western cultural experiences[53].

“Therapeutic support is not always culturally or individually accessible to people. It might not always be the way people want to, or feel able to, deal with difficulties. It often comes from a Western perspective and that doesn’t work for everybody.” (i10)

The literature identified explicit consideration of therapeutic boundaries as important[68]. Mental healthcare providers working with potentially vulnerable groups such as ASR experience many challenges. ASRs may have undergone traumatic events that move providers beyond compassion and empathy towards secondary trauma. Additionally, providers without appropriate training may be at greater risk of overstepping boundaries of the therapeutic relationship [68]. The British Psychological Society outlined respect, responsibility, integrity, and competence as four pillars to guide ethical therapeutic practice [70]. Such guidelines can support practice in this area of work.

The literature showed treatment modalities as important in managing mental health among ASR [60, 71]. Given the prevalence of trauma among ASR and the relative lack of specialised services in the UK, literature and interviewees supported development and strengthening of trauma psychotherapy for ASR [69]. All interviewees indicated new psychotherapeutic approaches were needed, as NHS psychological treatment is *“one size fits all”* using short-term cognitive-behavioural therapy not appropriate for many trauma survivors. Additionally, NHS psychotherapeutic support is limited to ten sessions and as one interviewee noted, “*they may need at least 12 sessions*” *(i8).* For some ASR, talk-therapies can cause reliving rather than resolving past experiences, which can be harmful without experienced trauma-informed support [53, 64]. Reflective practice, to help manage dissociative symptoms through identifying triggers and practicing grounding techniques, shows some promise for ASR clients [72]. Other suggestions included sleep therapies or activity-based therapies for those with trauma symptoms [71].

### Social support for mental health as an enabler

Literature and interviewees indicated some mental health service needs could be reduced by better social support. Both described the psychological trauma ASR may experience arriving in the UK in hope of a new life [54], but instead encountering a hostile environment and difficulties with general living that affect their mental wellbeing [60]. Better living conditions and ending hostile environment policies could reduce some pressures on mental healthcare services.

“…so, I think practical problems have a big impact on mental health to the point where it can produce crises where there may not have been otherwise… Immediate needs may need attention before it’s appropriate to look at those deeper things” (i10)

Considerable mental distress among ASR stems from personal-level and community-level factors including living-conditions, cultural adaptation, and financial needs [9], alongside structural factors such as difficult asylum processes and structural violence [73]. Mental disorders may originate prior to arrival or originate in the circumstances in which ASR find themselves [16, 60]. In the latter case, practical difficulties should be addressed, rather than focusing on psychotherapeutic support alone [16].

“I’ve certainly seen counsellors, through IAPT, documentation come back essentially saying this persons’ mental health needs are entirely at a practical and social level, but they didn't feel that counselling or therapy of any kind would be useful as their anxiety was entirely in concerns about destitution and immigration.” (i3)

Majumder described interviews with unaccompanied refugee minors indicating that focusing on overcoming current living problems helped them mitigate prior traumas. Young or unaccompanied ASR may need safety and stabilisation before considering therapies.

“Trauma focused CBT or EMDR, there's 2 or 3 different recommended treatments, but they should not offer that straight away to newly-arrived unaccompanied and separated young people, because they need something very different. They need stabilisation, they need help with their sleep because that's been completely dysregulated by the experience.” (i1)

### Supportive policies as enablers

Except for England, healthcare access is still free of charge even for rejected asylum-seekers in the UK [14, 29, 30, 32]. Policies such as unrestricted access to free primary and emergency care and not withholding treatment that is ‘urgent or immediately necessary’ because someone cannot pay for it, are enablers to mental healthcare access [50]. Interviewees supported this, suggesting that refusing care for undocumented migrants could pose additional health system pressures if they go untreated:

“A patient […] was refused secondary care for some months and then we got him asylum support on medical grounds […]. During that time it got to a crisis point where he was sectioned… If they dealt with it within the secondary care system when they got the appropriate support at the right time, that emergency situation hopefully wouldn't have happened.” (i3)

In a positive policy reversal, the Home Office abolished NHS data sharing for immigration tracking in 2018 [41]. However, not all ASR may be aware of this and still fear attending services if information on this reversal is not circulated.

## Discussion

### Key findings

This scoping review provides an opportunity to examine current literature evidence and discussion on this important and under-researched topic. Interviews added rich experiential data from service providers and advocates trying to support ASR, to clarify and help validate literature findings. Literature and interview sources found that while needs for mental health support among ASR in the UK are significant, both access to and quality of mental healthcare for ASR is lacking. Both described the mental health impacts of migration and post-migration stressors and the barriers to mental healthcare and support, particularly due to policy and legal constraints. They also offered some potential enablers, including supportive policies and psychotherapeutic approaches [10, 16].

While inequalities in access to primary care are incompatible with NHS guidance or the Equality Act (2010) [74], given years of austerity, politics around Brexit, and struggles to provide basic services, it is perhaps not surprising that ASR mental health needs are not prioritised. The hostile environment created by tightened immigration laws and tracking has increased fear and reduced trust among ASR [41, 59, 64]. This overlaps with health system constraints due to chronic underfunding (e.g. excess workload, salary freezes, staff shortages) and increasingly restrictive health policies, such as user-fees, which contributes to a lack of will to provide additional specialist services (e.g. interpreters) [16]. Recent NHS charging policies are not well understood by ASR [46] or even by all health- workers, leading to inconsistencies across the UK.

### Implications for policy, practice, and further research

Findings indicate many mental health problems experienced by ASR in the UK could be improved if socio-cultural and political-economy barriers could be addressed, including the hostile environment, increasing constraints due to Brexit, and provider attitudes [41–43]. The current UK policy environment provides very limited potential enablers [50]. However, in the interests of supporting existing evidence, we advocate that the UK government consider the following. First, ending secondary care charging in England to provide a united devolved response for all UK regions. Second, improving contributory factors to mental distress and illness, such as housing and financial stability for ASR families, to reduce extra burden on the NHS. Third, implementing culturally-appropriate treatment and piloting reflective practice, and adequate health-worker training and interpreters to improve the effectiveness of service provision for ASR. ASRs desire to connect with providers with similar backgrounds or experiences [63] could be a potential platform or bridge to expand service capacity by employing ASR to support mental health services for their communities.

Practitioners require evidence-based clinical recommendations to supporting ASR mental health. While government policies related to resettlement and healthcare access for ASR exist [16], mental healthcare is not specifically outlined beyond action plans and recommendations [29–35, 37, 38] that vary across the UK regions [16]. As resettlement numbers rise, funding should increase to enable equitable resettlement and living conditions. Poorer environmental conditions increase the likelihood of lower psychological wellbeing [60, 75], and ultimately strain the statutory services providing mental healthcare. There is a cost–benefit argument for providing social support for practical difficulties and environmental adjustments that worsen psychological wellbeing for ASR [16]. Better wellbeing, for both ASR and British citizens, will lower the strain on the NHS. A top-down approach is needed for concrete change within the UK, to reduce legal constraints and create a more positive environment for everyone.

Additional rigorous research is clearly needed. The literature on ASR mental healthcare in the UK is limited and primarily qualitative, with many study samples (n = 16) under twenty people. Gaps in the literature include specific mental healthcare needs and access, inclusion of a broader age range in qualitative research, differences in needs and barriers across countries-of-origin and asylum experience, and assessment of treatment approaches for ASR. While the literature indicates general healthcare access among ASR is inconsistent [24, 50, 52, 55] and suggests restructuring the asylum-seeking process and providing adequate housing would support mental health needs [63, 67, 76], data on access for specific mental health needs or services is lacking. This indicates a need for larger-scale research on ASR mental health needs and services provision. Age ranges and countries-of-origin of ASR varied in the literature. Unaccompanied children and adolescents were most studied [45, 53, 66, 71, 77, 78], accounting for eight different study samples, suggesting more research on the lived experiences of men and women is needed. Research is needed on whether ASR from different countries, or those who have been granted or refused asylum, experience additional needs or barriers [24], and on identifying and assessing culturally-relevant psychotherapeutic treatments [79]. A stronger evidence base would encourage policymakers and practitioners to implement these practices across the UK [79].

### Limitations

Several limitations should be considered. First, time and were funding limited, reducing potential depth. Scoping reviews, by definition provide a broad topical overview. However, inclusion of interviews helped ameliorate this by providing rich data. Second, one author conducted most work as part of her MSc studies. Single investigator searching and screening is also acceptable for scoping reviews, given their more straightforward exclusion criteria than systematic reviews, but may have affected some topics [80]. Third, quality appraisal of individual papers, though not required for scoping reviews, was not undertaken due to time and resource constraints. Findings may thus differ from reviews that excluded sources based on type or quality appraisal [81]. Fourth, interviewees were ASR mental healthcare service providers from England, rather than UK-wide, reducing in-depth examination of services in Wales, Scotland, and Northern Ireland. A larger stakeholder sample would have provided more useful data, while inclusion of commissioners, policymakers and ASR themselves would have provided additional perspectives. Future research with stakeholders should include these additional perspectives and all devolved regions of the UK.

## Conclusions and recommendations

Mental health problems are common among ASR, particularly because the reason for seeking asylum usually involves one or more traumatic life episode [10, 11]. Additionally, the asylum- seeking process causes additional anxieties because of: (i) uncertainty about immigration status and finding a new home; (ii) minimal or no access to funding support, particularly for refused asylum- seekers; (iii) lack of clarity about rights to work or inability to work until immigration status is received; (iv) uncertainty about access or costs of healthcare, making mental healthcare access even more difficult; (v) language barriers and overstretched healthcare professionals; and (vi) adapting to a society which may be hostile to their needs. UK government policy could do more to address these issues. This would help ASR begin healing from the traumas they suffered so they can contribute fully in their new society without the added burdens of unresolved and ongoing traumas. A less hostile environment towards ASR would allow for their easier integration and contributions to British society.

## Supplementary Information

**Additional file 1. **Table of Charted Data. Data was charted using the following fields: lead author, publication year, location, source type, title, aims, methods, population, and key findings.

**Additional file 2.** PRISMA-ScR Checklist.

## Data Availability

Datasets collected and analysed during this study are available from the corresponding author on reasonable request.
