# Supplementary material for: Mental healthcare for asylum-seekers and refugees residing in the United Kingdom: a scoping review of policies, barriers, and enablers
Source: Int J Ment Health Syst. 2021 Jun 14;15:60. doi: 10.1186/s13033-021-00473-z (PMC8201739; doi:10.1186/s13033-021-00473-z)
Supplement: Supplementary file 1 — Additional file 1. Table of Charted Data. Data was charted using the following fields: lead author, publication year, location, source type, title, aims, methods, population, and key findings. [file 13033_2021_473_MOESM1_ESM.docx]

**Additional File 1. Table of charted data**

| **Lead author** | **Publication Year** | **Location** | **Source Type** | **Methods and populations** | **Key findings** |
| --- | --- | --- | --- | --- | --- |
| Brandenberger & Tylleskar[1] | 2019 | Western countries including UK | Research article | Systematic literature review of quantitative and qualitative studies between 2000 and 2017 of current knowledge on health care delivery to migrants and refugees in high-income countries from multiple perspectives. 35 articles were included from US, Australia, Europe and the UK. | Thematic analysis found three main challenges in health care delivery: Communication, continuity of care, and confidence. This structure gives a patient-centred summary of key challenges in healthcare. |
| Brenman[2] | 2020 | London, UK | Research article | Qualitative ethnography of ‘embodied belonging’ in London intercultural psychotherapy centre, including sociomaterial analysis of ethnographic material and visual data from mapping interviews with 2 participants. | This study found how aspects of place became entangled in client experiences of access: waiting, client group and deservingness of care. |
| Brooks[3] | 2019 | UK | Clinical practice article | Review of reflective practice with refugees, asylum-seekers, and survivors of torture; and the structure of critical incident analysis skills, no study population. | 5 steps to improve clinical practice of IAPT CBT therapists who work with diverse populations. Complexities of practice include language barriers, increased prevalence to PTSD and comorbid mental health problems. There is risk of burnout for therapists who work in this area. Reflective practice can improve clinical practice with this population. |
| Chiarenza *et al*[4] | 2019 | EU countries, UK | Research article | Mixed methods study with:   1. 20 semi-structured interviews and 10 focus groups in 10 EU countries (Greece, Italy, Spain, Slovenia, Hungary, Austria, Belgium, Denmark, the Netherlands, UK) with 128 healthcare providers to understand challenges relating to the refugee crisis. 2. Systematic literature review of 251 sources on barriers and solutions to healthcare access for asylum-seekers and refugees. | The study found solutions are aimed at responding to emergencies rather than structural changes in the health system. |
| Cowles & Griggs[5] | 2019 | England, UK | Research article | Qualitative case study of a woman with a severe trauma history seeking asylum in England, to explore how work with people who are seeking asylum can involve creating containment by having boundaries and addressing psycho-social issues by crossing boundaries. | The therapeutic relationship and careful boundaries of inflicting harm. Self-reflection, supervision, critical thinking and discussion with the service-user can help guide appropriate boundary management. |
| Cox[6] | 2020 | UK, England And Wales | Commentary | Analysis and critique of the documents and policies that influenced the Green Paper, no study population | Findings suggest potential benefit of refugee children and young peoples future mental health and well-being. The paper is focused on western-centric models in its understanding of refugee experiences and the management of trauma and mental health. It does not recognise the meanings and significance of culture and diversity to facilitate engagement and support of young people with mental health issues. |
| Crawshaw &Kirkbride[7] | 2018 | UK | Short communication | Review and formal evaluation of Migrant Health Guide, no study population | The PHE migrant health guide is recognised as valuable resource for healthcare professionals. The relaunched guide in 2017 builds on the previous version in raising awareness of the main issues and providing evidence- based advice to improve the health of migrant and refugees in the UK. |
| Doctors of the world[8] | 2015 | England, UK | Agency report | Case study of a 23 year old woman from Vietnam. Record of 849 DOTW attempts to register patients with NHS GPs in England between 4 March and 21 October 2015. | The purpose was to review the accessibility of GP registration for vulnerable people living in the England. The barriers impact the vulnerable groups in the UK. GP practices should be trained on entitlements to NHS care and working with vulnerable groups. |
| Fang *et al*[9] | 2015 | Manchester, UK | Research article | Qualitative study with 35 in-depth interviews and 5 focus groups with Somali and Iraqi refugees, asylum-seekers, and those applying for support to explore health and healthcare experiences through personal accounts. | Barriers to accessing health care was associated with social disadvantage and restrictions of the welfare system. Re-evaluation of the asylum seeking process is required to improve the situation. |
| Hiam *et al*[10] | 2018 | UK | Perspective piece | Review of UK Government made public a Memorandum of Understanding (MoU) between the Department of Health, National Health Service (NHS) Digital and the Home Office. No study population | Fears of data sharing and hostility from the government towards migrants. There are strong grounds to believe that the MoU threatens individual and public health, while placing health professionals in a difficult position. This paper supports calls to suspend it until there is a detailed consultation of its impacts. |
| Hiam *et al*[11] | 2019 | UK | Research article | Workshop proceedings of the First World Congress on Migration, Ethnicity, Race and Health. No study population | The workshop documented how migrants face many diverse barriers in accessing healthcare. Civil society organisations can challenge and overcome structural barriers to care. |
| Jannesari & Molyneaux[12] | 2019 | UK | Research article | Qualitative study with 3 face-to-face interviews with people seeking asylum in 2005-2017 from Africa lasting 3-5 hours, to understand how people seeking asylum make sense of their migration experience and the factors impacting the mental health of people seeking asylum in the UK. | Interviewees felt hostile environment which made it difficult to recover from trauma. Policies relating to the asylum process negatively affected their health and wellbeing. |
| Juárez *et al*[*1*3] | 2019 | Europe, UK included | Research article | Systematic review of studies 2000-2017 comparing the health effects of non-health-targeted public policies on migrants with those on a relevant comparison population in high-income countries. 46 articles narratively synthesised and 19 for meta-analysis. | Restricted entry and integration policies were related to negative effects on migrant health outcomes in high income countries |
| Kang *et al*[14] | 2019 | UK | Research article | Qualitative study with face-to-face semi-structured interviews with 18 refugees and asylum-seekers from Pakistan, Syria, Iran, Libya, Eritrea, Sudan, Guinea, and Ivory Coast accessing primary healthcare in the UK in 2018. | Thematic analysis found a lack of understanding in the NHS structure and navigating systems for asylum seekers and refugees. Multiple barriers were identified for this population group accessing primary healthcare including inadequate interpretation and discrimination at GP surgeries. |
| Karamanidou *et al*[15] | 2020 | England, Ireland, Scotland, Wales,  UK | Research report | Qualitative content analysis approach with:   1. 15 interviews conducted by the RESPOND UK teams with migrants, and 16 interviews with meso-level stakeholders 2. Draws on legal texts, policy documents, policy guidance issued by the Home Office. It also draws on reports by NGOs and human rights organisations and relevant academic research and literature. | This is focused on reception policies, practices, and humanitarian responses to the current refugee crisis. The UK reception system is oriented toward migration control rather than providing for the needs of asylum seekers. |
| Majumder *et al* [16-18] | 2015/2019 | England, UK | Research article | Qualitative study, semi-structured interviews with 15 unaccompanied refugee children age 15-18yrs and their carers from Afghanistan, Iran, Eritrea, and Somalia. To explore their experiences, perceptions and beliefs of mental illness, focusing on stigma, their opinions of mental health services and treatments, and perceptions of barriers to using mental health services.. | 3 themes were identified: negative perceptions of the concept of mental illness, anticipated social implications of suffering form mental illness, and denial of mental illness. Participants suggested cultural competency and sensitivities are needed when making assessments.  Suppressing trauma and focusing on present day issues was labelled as the best way of coping.  Many held negative attitudes toward mental health and had a lack of trust in services. |
| McKeown *et al*[19] | 2020 | UK | Editorial | No study population | Highlights the public health hazards due to the governments treatment of migrants and locate within the winder context of mental health. |
| Mohamed[20] | 2012 | UK | Thesis | Mixed-methods study with:   1. Literature review of child mental health and psychological wellbeing 2. Questionnaires and semi-structured 24 interviews of school children 9-19 years and parents from Afghanistan, Uganda, Somalia, Albania, Congo, Ethiopia, and Pakistan. | Bullying and racial harassment outlines as an issue for migrants in the UK. Resilience was found to be a factor of positive adaptation of refugee children and the psychological well-being |
| Murphy *et al*[21] | 2020 | UK | Research article | Mixed-methods digital survey of 220 children’s health practitioners working in the UK on attitudes towards and understanding of UK healthcare charging. Recruitment was through email bulletins, and social media platforms (twitter) | Understanding the NHS charging regulations and impacts on migrants healthcare access. Most participants were not confident in interpreting the charging regulations. |
| Murphy & Vietan[22] | 2017 | Northern Ireland, UK | Research report | Mixed-methods study with:   1. Literature review of research and policy developments 2. 78 short questionnaires completed by service providers, organisations, and local councils. 3. 25 semi-structured interviews and 2 focus groups (1 with 10 participants, and 1 with 3) with service providers, voluntary and charitable organisations, and members of asylum seeking and refugee communities in Northern Ireland. | Commissioned by the Racial Equality Unit in the Executive Office to inform the integration strategy for Northern Ireland. Participants identified a range of challenges that needed to be addressed including housing, health, employment, education and legal issues to improve the pathway to integration. Participants identified a need for a refugee integration strategy to be developed in NI. |
| Nellums *et al*[23] | 2018 | UK | Research report | Qualitative study of one to one conversations and small group discussions with 21 people (17 women and 13 men) seeking or refused asylum, plus healthcare professionals who lived in Glasgow, Nottingham, Swansea, and London. | The report examines the barriers and enablers experienced by people seeking or refused asylum, when they try to use health services in Britain. The findings highlight that there are clear barriers to accessing healthcare both at systemic policy level and in implementation and practice. Participants highlighted the importance of a patient-centred approach to providing healthcare. |
| Nellums *et al*[24] | 2018 | UK | Research report | Mixed-method study, with:   1. Narrative literature review, including 26 sources between 2014-2018 2. Data from DOTW clinics used to identify barriers. | Summary of the policy and legislative context shaping people’s healthcare entitlements and experiences of accessing healthcare. Two main barriers were those resulting from legislation and policy, and those arising from everyday practice from services. |
| Patel, Corbett, DOTW[25] | 2017 | England, UK | Case study report | Case study of a 27 year old man from Sri-Lanka and analysis of 1,717 DPTW attempts to register patients with GPs between January and December 2017. | One fifth of cases were refused. The barriers to registration observed indicate worrying inequities to primary care. This research recommends that GP practices and clinical staff receive training on entitlements to care. |
| Piacentini *et al*[26] | 2019 | Scotland, UK | Research article | Qualitative study. Individual in-depth interviews and group discussions with 19 research participants in Glasgow: including, interpreters, health care providers and service users. Interviews focused on current practice, coping strategies and knowledge of working in multilingual contexts. | Asylum seekers have less knowledge about navigating systems as they cannot access information beforehand. There is a need to widen out axes of inequality in healthcare encounters to include migration-related variables. |
| Poduval *et al*[27] | 2015 | London, UK | Research article | Qualitative study with semi-structured interviews of 16 undocumented migrants and 4 volunteer staff at a charity clinic from Afghanistan, Bangladesh, Brazil, China, India, the Philippines, Romania, Sri Lanka, and Uganda to explore the experiences of undocumented migrants trying to access primary care in the UK | Findings suggest there is little understanding of healthcare entitlements for migrants. Migrants said they would not be able to afford charges to primary care. More research is needed to inform effective policy. |
| Priebe *et al*[28] | 2016 | European region, including UK | Research article | Systematic review including 69 academic and grey sources on policies and interventions that improve mental healthcare for refugees, asylum-seekers, and irregular migrants. | Refugees, asylum seekers and irregular migrants are exposed to risk factors for mental disorders before, during, and after migration. The rates of post-traumatic stress disorder in refugees and asylum seekers are higher, depression and anxiety is likely to increase over time and poor socioeconomic conditions contribute to mental wellbeing |
| Quinn[29] | 2014 | Scotland, UK | Research article | Participatory action research with 10 focus groups with 100+ asylum-seekers and refugees in Scotland from Somalia, Eritrea, Pakistan, Iran, Iraq, China and Sri Lanka to explore participant views on mental health problems, stigma and discrimination during focus group discussions. | The study outlines help-seeking issues, inability to trust foreign providers and poor attitudes to mental health. It confirms existing literature on how migration can have adverse effects on mental health and wellbeing. |
| Rae[30] | 2014 | London, UK | Thesis | Qualitative study with semi-structured interviews with 12 Somali male refugees, and 3 focus groups discussions to explore how Somali male refugees in the UK understand and perceive the Western concept of depression, alongside their views on coping and professional help in the UK. | Fear of stigma from their community towards mental health was noted, as well as language barriers. Depression was a result of difficulties associated with migration and disconnection. |
| Riza *et al*[31] | 2020 | Europe, UK | Research article | Scoping review including a systematic search finding 118 academic sources on effective community-based healthcare models and interventions for migrants and refugees | Concluded 15 best practices in community-based healthcare. Training for healthcare professionals to work with the population group, interventions for mental health and culturally and linguistically adapted provisions. |
| Robertshaw *et al*[32] | 2017 | High income countries (UK) | Research article | Systematic review of 26 articles including 21 primary qualitative studies with 357 participants exploring challenges and facilitators for health professionals providing primary healthcare for refugees and asylum-seekers in high-income countries. | 11 descriptive themes included that trust is vital for good healthcare delivery, as well as cultural competence and communication between healthcare professional and patient. Professionals should be sufficiently resourced to meet the complex needs of refugees and asylum seekers. |
| Sanchez-Cao. *et al*[33] | 2013 | London, UK | Research article | Mixed-methods study including:   1. Socio-demographic data on 71 UASC residing in London, predominantly black African males age 17 years 2. Self-reported questionnaires on trauma events, general psychological distress, post-traumatic stress symptoms, depressive symptoms and contact with mental health services. | Findings show 47 were at risk of post-traumatic stress disorder and 9 high risk of depressive disorder. Only 12 were in contact with services, and related to depression rather than PTSD. Reasons for lack of service utilisation include language difficulties, limited awareness of services, and difficulty registering with GPs. |
| Satinsky *et al*[34] | 2019 | Europe, UK specifics | Research article | Systematic review including 27 articles from 2007-2017 with studies that took place in 18 EU countries, to examine evidence on Mental health and psychological support (MHPSS) service utilisation and access among refugees and asylum-seekers. | Articles showed inadequate MHPSS utilisation. Problems with navigating systems arose, and more ethnic minority staff were wanted within psychiatric services to aid building trust. |
| Strang & Quinn[35] | 2019 | UK | Research article | Qualitative study including 30 male Iranian and Afghani refugee participants in 4 participatory workshops initially individually recruited and through posters with further snowballing recruitment. | The indicators of integration framework, a conceptual framework defining core domains of refugee integration. Patterns of trust were key for integration, migrants felt isolation and loss of identity. Results enhances our theoretical understanding of refugee social connections. Findings showed men had few social connections, it confirms a pattern of extreme social isolation and poor access to services and rights. |
| Taylor[36] | 2018 | UK | Policy report | Policy Debate, briefing prepared for the debate in the House of Lords, on the motion moved by Lord Bassam of Brighton, no study population | This report summarises the background to the policy, measures connected to it, commentary on the impact of the policy, and recent developments. Commentators have claimed that the policy measures can cause racism and discrimination. |
| Tesfaye & Day[37] | 2015 | England, UK | Research article | Qualitative pilot study including semi-structured interviews with 8 health visitors asked about perceptions of barriers to health and wellbeing faced by European migrant families and the common challenges in practice. | This research identified significant barriers to the health and wellbeing of European Migrant Families in a Merseyside borough. Barriers included wider determinants of health and barriers in accessing health, public and community services. Current local and national intelligence systems are not able to quantify needs, inequalities and service use by migrants. |
| Viner[38] | 2018 | England & Wales, UK | Research report | Mixed methods:   1. Literature search on the mental health needs of unaccompanied young people and policy recommendations. 2. Freedom of information requests to department of education on SDQ records for looked after children 3. In-depth interviews with 10 unaccompanied young people age 16-25 years and 10 mental health professionals to provide an overview of what is currently known about the mental health needs of unaccompanied young people living in England and Wales. | Findings: the effects of traumatic events that young people have faced prior to arrival in the UK can continue to affect them for substantial periods of time, and their mental health can deteriorate if they face barriers in settling their lives and their long-term prospects. Expansive structural change is required in order to build sensitivity to unaccompanied young people’s needs in systems that support them. |
| Waterman e*t al*[39] | 2020 | England, UK | Case study praxis | Case study using a fictitious case of an asylum-seeker from Sri Lanka presenting to a mental health service in England to highlight some of the difficulties in assessing and treating this patient group and providing advice to clinicians on clinical and practical management. | There remains ethical and legal issues that need addressing nationally, including the sharing of patient data between NHS series and the Home Office, including the upfront charging for NHS services. Urgent assessment is needed into the impact on vulnerable groups of extending charging into NHS communities. |

1. Brandenberger J, Tylleskär T, Sontag K, Peterhans B, Ritz N. A systematic literature review of reported challenges in health care delivery to migrants and refugees in high-income countries-the 3C model. BMC Public Health. 2019;19(1):755.

2. Brenman NF. Placing precarity: access and belonging in the shifting landscape of UK mental health care. Culture, medicine and psychiatry. 2020.

3. Brooks M. The importance of using reflective practice when working with refugees, asylum seekers and survivors of torture within IAPT. The Cognitive Behaviour Therapist. 2019;12.

4. Chiarenza A, Dauvrin M, Chiesa V, Baatout S, Verrept H. Supporting access to healthcare for refugees and migrants in European countries under particular migratory pressure. BMC health services research. 2019;19(1):513.

5. M Cowles MG. Considering boundaries when doing therapeutic work with people who are seeking asylum: a reflective case study. . British Journal of Guidance & Counselling 2019(1):50-64.

6. Cox P, McDonald JM. Analysis and critique of 'Transforming children and young people's mental health provision: A green paper': Some implications for refugee children and young people. Journal of child health care : for professionals working with children in the hospital and community. 2020;24(3):338-50.

7. Crawshaw AF, Kirkbride H. Public Health England's Migrant Health Guide: an online resource for primary care practitioners. Public Health. 2018;158:198-202.

8. World Dot. Registration Refused: a study on access to GP registration in England Report. 2015.

9. Fang ML, Sixsmith J, Lawthom R, Mountian I, Shahrin A. Experiencing ‘pathologized presence and normalized absence’; understanding health related experiences and access to health care among Iraqi and Somali asylum seekers, refugees and persons without legal status. BMC public health. 2015;15(1):1-12.

10. Hiam L, Steele S, McKee M. Creating a ‘hostile environment for migrants’: the British government’s use of health service data to restrict immigration is a very bad idea. Health Economics, Policy and Law. 2018;13(2):107-17.

11. Hiam L, Gionakis N, Holmes SM, McKee M. Overcoming the barriers migrants face in accessing health care. Public health. 2019;172:89-92.

12. Jannesari S, Molyneaux E, Lawrence V. What affects the mental health of people seeking asylum in the uk? A narrative analysis of migration stories. Qualitative Research in Psychology. 2019:No-Specified.

13. Juarez SP, Honkaniemi H, Dunlavy AC, Aldridge RW, Barreto ML, Katikireddi SV, et al. Effects of non-health-targeted policies on migrant health: a systematic review and meta-analysis. The Lancet Global health. 2019;7(4):e420-e35.

14. Kang C, Tomkow L, Farrington R. Access to primary health care for asylum seekers and refugees: a qualitative study of service user experiences in the UK. British Journal of General Practice. 2019;69(685):e537-e45.

15. Karamanidou L FJ. Reception Policies, Practices and Responses- UK Country Report. Glasgow Caledonian University; 2020.

16. Majumder P. Exploring stigma and its effect on access to mental health services in unaccompanied refugee children. BJPsych Bulletin. 2019(6):275-81.

17. Majumder P, Vostanis P, Karim K, O'Reilly M. Potential barriers in the therapeutic relationship in unaccompanied refugee minors in mental health. Journal of mental health (Abingdon, England). 2019;28(4):372-8.

18. Majumder P, O'Reilly M, Karim K, Vostanis P. 'This doctor, I not trust him, I'm not safe': the perceptions of mental health and services by unaccompanied refugee adolescents. Int J Soc Psychiatry. 2015;61(2):129-36.

19. McKeown M, Dropkin G. Migrant health charges: a scandal amidst the crisis. Journal of psychiatric and mental health nursing. 2020.

20. Mohamed S. The mental health and psychological well-being of refugee children: an exploration of risk, resilience and protective factors University of East London; 2012.

21. Murphy L BJ, Hopkinshaw B, Boutros S, Russell N, Firth A, McKeown R, Steele A. . Healthcare access for children and families on the move and migrants. BMJ Paediatrics Open. 2020(1).

22. Murphy F, Vieten UM. Asylum seekers and refugees’ experiences of life in Northern Ireland. Belfast: The Executive Office. 2017.

23. Nellums LB RK, Hargreaves S, Friedland J, Miller A, Hiam L, Le Deaut D. . The lived experiences of access to healthcare for people seeking and refused asylum. Imperial College London, Doctors of the World; 2018.

24. Nellums LB RK, Hargreaves S, Friedland J, Miller A, Hiam L. Access to healthcare for people seeking and refused asylum in Great Britain: a review of evidence. Imperial College London, Doctors of the World UK; 2018.

25. A Patel JC, Doctors of the World. Doctors of the World: Registration Refused: a study on access to GP registration in England. 2017.

26. Piacentini T ODC, Phipps A, Jackson I, Stack N. . Moving beyond the ‘language problem’: developing an understanding of the intersections of health, language and immigration status in interpreter-mediated health encounters. Language and Intercultural Communication. 2019(3):256-71.

27. Poduval S HN, Jones L, Murwill P, McKee M, Legido-Quigley H. . Experiences among undocumented migrants accessing primary care in the United Kingdom: a qualitative study. International Journal of Health Services. 2015(2):320-33.

28. Priebe S, Giacco D, El-Nagib R. Public health aspects of mental health among migrants and refugees: a review of the evidence on mental health care for refugees, asylum seekers and irregular migrants in the WHO European Region: World Health Organization. Regional Office for Europe; 2016.

29. Quinn N. Participatory action research with asylum seekers and refugees experiencing stigma and discrimination: The experience from Scotland. Disability & Society. 2014;29(1):58-70.

30. Rae S. Somali male refugees: Perceptions of depression and help-seeking University of East London; 2014.

31. Riza E, Kalkman S, Coritsidis A, Koubardas S, Vassiliu S, Lazarou D, et al. Community-Based Healthcare for Migrants and Refugees: A Scoping Literature Review of Best Practices. Healthcare (Basel). 2020;8(2).

32. Robertshaw L, Dhesi S, Jones LL. Challenges and facilitators for health professionals providing primary healthcare for refugees and asylum seekers in high-income countries: a systematic review and thematic synthesis of qualitative research. BMJ open. 2017;7(8).

33. Sanchez-Cao E, Kramer T, Hodes M. Psychological distress and mental health service contact of unaccompanied asylum-seeking children. Child: care, health and development. 2013;39(5):651-9.

34. Satinsky E, Filippou TA, Kousoulis AA. Multiculturalism and Compassion: Responding to Mental Health Needs Among Refugees and Asylum Seekers Comment on "A Crisis of Humanitarianism: Refugees at the Gates of Europe". Int J Health Policy Manag. 2019;8(12):734-6.

35. AB Strang NQ. Integration or Isolation? Refugees’ Social Connections and Wellbeing. Journal of Refugee Studies. 2019.

36. Taylor S, Debelle G, Modi N. Child refugees: The right to compassion. BMJ (Online). 2016;355:i6100.

37. Tesfaye HT, Day J. Health visitors' perceptions of barriers to health and wellbeing in European migrant families. Community practitioner : the journal of the Community Practitioners' & Health Visitors' Association. 2015;88(1):22-5.

38. Viner et al. Distress signals: unaccompanied young people’s struggle for mental health care, The Children’s Society. 2018.

39. Waterman LZ, Katona C. Assessing asylum seekers, refugees and undocumented migrants. BJPsych Bulletin. 2020;44(2):75-80.
